# Cardiovascular risk factors and cardiac dysfunction in people with HIV and breast cancer: an observational cohort study in Botswana

**DOI:** 10.1186/s40959-025-00417-3

**Published:** 2026-01-22

**Authors:** Henrietta Afari, Congying Xia, Julius Mwita, Thato Moshomo, Anran Huang, Maliha Shaikh, Sebaga W. Motlhwa, Tlotlo Ralefala, Peter Vuylsteke, Scott Dryden-Peterson, Priscilla Y. Hsue, Robert Gross, Mosepele Mosepele, Bonnie Ky, Yehoda M. Martei

**Affiliations:** 1 Division of Cardiology, Department of Medicine, Perelman School of Medicine, University of Pennsylvania, 3400 Civic Center Boulevard, Philadelphia, PA 19104 USA; 2 Princess Marina Hospital, Gaborone, Botswana; 3 University of Botswana, Gaborone, Botswana; 4 Vrije Universiteit Brussel, Ixelles, Belgium; 5 Brigham and Women’s Hospital, Harvard T.H. Chan School of Public Health, Boston, MA United States; 6 Botswana Harvard Health Partnership, Gaborone, Botswana; 7 Division of Cardiology, Department of Medicine, University of California, Los Angeles, CA USA; 8 Division of Infectious Diseases, Department of Medicine, Perelman School of Medicine, University of Pennsylvania, Philadelphia, USA; 9 Division of Hematology - Oncology, Department of Medicine, Perelman School of Medicine, University of Pennsylvania, Philadelphia, USA

**Keywords:** HIV, Breast cancer, Anthracyclines, Trastuzumab, Hypertension, Cardiovascular disease, Botswana, LVEF

## Abstract

**Background:**

HIV, cancer, and their respective treatments are independently associated with cardiovascular risk, but limited data exist on the intersection of these conditions. The purpose of this study was to gain insights into the cardiovascular risk factor burden and cardiac function in people with HIV (PWH) treated for breast cancer.

**Methods:**

In a cohort of PWH and breast cancer treated with anthracyclines and/or trastuzumab (2017–2022) in Botswana, we assessed pre-treatment (baseline) left ventricular ejection fraction (LVEF), and prospectively obtained an echocardiogram at least one year after cancer treatment initiation. Wilcoxon signed rank sum test was used to test the differences between baseline and follow-up LVEF.

**Results:**

Thirty-three women were enrolled at a median of 2.1 years (Quartile (Q)1-Q3 1.8–3.1) from their cancer treatment initiation. The median age was 48.0 years (Q1-Q3 44.0–54.0). All but one patient was on antiretroviral therapy (ART); the median ART duration was 11.6 years (Q1-Q3 6.3–15 years) with a median viral load of 30 (Q1-Q3 0–30) and CD4 count of 874 (Q1-Q3 361–1131). At baseline, 70% were obese or overweight, and 24.2% reported hypertension; this increased to 30.3% at follow-up. The median LVEF at baseline was 65% (Q1-Q3 60–68%), and decreased to 62% (Q1-Q3 59–65%) at follow-up; an absolute difference of 2.9%, 95%CI: -5.3 to -0.2% (*p* = 0.038). There was no report of clinical heart failure.

**Conclusions:**

Obesity and hypertension are highly prevalent amongst PWH and breast cancer. We also noted a statistically significant, but modest decline in LVEF after cancer therapy initiation. Further studies are needed to prospectively characterize the cardiovascular risk factor burden and changes in cardiac structure and function following cardiotoxic cancer treatment in this population.

**Supplementary Information:**

The online version contains supplementary material available at 10.1186/s40959-025-00417-3.

## Introduction

Antiretroviral therapy (ART) has improved survival among people with HIV (PWH), resulting in an aging HIV population, with an estimated 8.1 million PWH older than 50 years [1]. This has led to an increase in age-related co-morbidities, including non-AIDS defining cancers such as breast cancer and cardiovascular (CV) disease [2, 3].

PWH also have a greater than 2-fold increased risk of CV disease compared to people without HIV [4], and an increased risk of CV complications, including a >3-fold increase in heart failure [5]. The increase in CV comorbidities is multifactorial including increased incidence of traditional CV risk factors, side effects from ART, and chronic inflammation from HIV itself.

Highly effective breast cancer therapies, including anthracyclines (e.g. doxorubicin) and HER2 + targeted therapies (e.g. trastuzumab) are widely used in the curative setting and have led to important survival gains among patients with breast cancer [6]. However, doxorubicin and trastuzumab carry an established risk of cancer therapy-related cardiac dysfunction and heart failure with reduced ejection fraction (HFrEF) which occurs in 10–15% of breast cancer patients [7, 8]. Detailed cardiac phenotyping in non-HIV cohorts with breast cancer showed a mean decrease in left ventricular ejection fraction (LVEF) of 3.6% at 1 year in patients who received doxorubicin alone [7], and a decrease of 6.6% at 1 year in patients who received both doxorubicin and trastuzumab [7]; The development of cardiac dysfunction in the short-term results in dose interruptions, treatment delays; and worse oncologic outcomes in the long-term [9, 10]. In the general population, traditional cardiovascular risk factors such as advanced age, hypertension, hyperlipidemia, obesity, and tobacco use are associated with increased risk for cardiac dysfunction following cancer therapy.

Cohort studies across the US and multiple countries in Africa have shown that PWH diagnosed with breast cancer have worse survival compared to breast cancer patients without HIV [3, 11, 12]. The underlying mechanisms for the observed survival disparities are poorly understood.

PWH and breast cancer may have an elevated risk of cardiac dysfunction following cancer-directed therapy given overlapping risk factors for CV disease from HIV and breast cancer therapy. However, no studies have examined cardiac dysfunction following anthracycline and HER2 + targeted therapies in PWH. We aimed to understand the cardiovascular risk factor burden and change in cardiac function in PWH receiving cardiotoxic breast cancer treatments.

## Methods

### Study population

We enrolled patients from a breast cancer cohort comprising patients with stage I-III breast cancer, who received curative-intent chemotherapy at Princess Marina Hospital, in Gaborone, Botswana. Females with HIV, ≥ 18 years of age, diagnosed with stage I-III breast cancer, who received doxorubicin and/or trastuzumab with a baseline echocardiogram with documented LVEF, and who initiated cancer treatment > 12 months prior were eligible to participate and approached for this study. Patients provided written informed consent. Patients who were pregnant or unable to consent were excluded. The study protocol was approved by the Institutional Review Boards at the University of Pennsylvania, the Botswana Human Research Development Committee, Ministry of Health, and Princess Marina Hospital.

### Echocardiography quantitation

Baseline echocardiograms had LVEF assessment performed by a single cardiologist at Princess Marina Hospital (J.M.). Repeat transthoracic echocardiograms were performed from August to December 2023 at Village Imaging Center in Botswana by a dedicated cardiac sonographer as part of this study. Measurements obtained included LVEF (using biplane Simpson’s method of discs), diastolic function (E/e’), LV end-diastolic (EDV) and end-systolic volumes (ESV), relative wall thickness, left ventricular end-diastolic dimension (LVEDD), right ventricular (RV) size and function and valvular function. All measurements were obtained as per the American Society of Echocardiography (ASE) guidelines [13].

### Clinical assessments

Clinical data, including CV and behavioral risk factors, were obtained using patient health records at baseline and follow-up. We collected data on cancer and HIV clinical characteristics and respective treatment histories including duration of ART. At baseline and follow-up, prevalent hypertension was assessed as a binary variable (yes/no). Additionally manual blood pressure measurements were obtained at the time of the repeat echocardiogram acquisition. Hypertension was defined as a blood pressure reading of 130/80 mm Hg or higher, according to AHA guidelines, and blood pressure categories were defined as follows: elevated blood pressures, 120–129/<80; stage 1 hypertension, 130–139/80–89; and stage 2 hypertension, ≥ 140/90 [14].

### Statistical analysis

Wilcoxon signed rank sum test was used to test the difference between baseline and post-cancer treatment measurement of LVEF, at least one year after cancer therapy initiation. A 2-sided p value < 0.05 was used to determine statistical significance. All analyses were conducted using R (Version 4.4, R Foundation for Statistical Computing, Vienna, Austria).

## Results

A total of 33 PWH and breast cancer who had documentation of a baseline echocardiogram enrolled in this cohort (Supplementary Fig. 1). Baseline HIV, cancer, and medical histories are summarized in Table 1. The median age was 48.0 (Quartile (Q)1-Q3 44.0–54.0) years. The median follow-up time from cancer treatment initiation was 2.1 years (Q1-Q3 1.8–3.1). All but one patient was on ART, and the majority (72.7%) were on an integrase inhibitor-based regimen, with an overall median duration of ART of 11.6 years (Q1-Q3 6.3–15). The median CD4 among those on treatment was 874 and the percent with a viral load (VL) < 30 was 50%. Overall, 97% of patients received anthracycline therapy, 12.1% received trastuzumab and 9.1% received both doxorubicin and trastuzumab. At baseline, prior to cancer treatment initiation, 24.2% of patients had a history of hypertension, 9.1% a history of tobacco use (oral, smoked or both), and 69.7% of participants were overweight or obese. The median LVEF at baseline was 65% (Q1-Q3 60–68%). None of the patients had congestive heart failure at baseline.

**Table 1:** Baseline clinical and demographic characteristics of study participants

		*N* = 33 (%)
**Demographic characteristics**		
Median age at enrollment, years	Median (Q1, Q3)	48.0 (44 to 54)
Marital status	Single	24 (73)
	Married	8 (24)
	Widowed	1 (3)
Education level	Junior Secondary	14 (42)
	Primary	7 (21)
	Tertiary	6 (18)
	Senior Secondary	2 (6)
	None	2 (6)
	Missing	2 (6)
Employment status	Employed	27 (82)
	Unemployed	4 (12)
	Missing	2 (6)
HIV characteristics*		
ART at enrollment		32 (97)
Median time on ART treatment, years	Median (Q1, Q3)	11.6 (6 to 15)
CD4 count	Median (Q1, Q3)	874 (361 to 1131)
Viral load copies per ml	Median (Q1, Q3)	30 (0 to 30)
ART regimen	Tenofovir, Lamivudine, and Dolutegravir	21 (64)
	Efavirenz, Emtricitabine and Tenofovir	5 (15)
	Abacavir, Lamivudine and Dolutegravir	2 (6)
	Nevirapine, Zidovudine and Lamivudine	2 (6)
	Emtricitabine, Tenofovir and Dolutegravir	1 (3)
	Emtricitabine, Tenofovir and Atazanavir	1 (3)
	Missing	1 (3)
**Cancer characteristics**		
Breast cancer stages at diagnosis	Stage II	12 (36)
	Stage III	21 (64)
Breast laterality	Left	19 (58)
	Right	13 (39)
	Bilateral	1 (3)
ER status	Pos	20 (61)
	Neg	13 (39)
PR status	Pos	12 (36)
	Neg	20 (61)
	Not done or missing	1 (3)
HER2 status	Pos	5 (15)
	Neg	26 (79)
	Not done or missing	2 (6)
Cancer Treatment	Doxorubicin	32 (97)
	Trastuzumab	4 (12)
	Doxorubicin + Trastuzumab	3 (9)
	Radiation therapy	20 (61)
**Medical history and risk factors**		
Tobacco history		3 (9)
History of hypertension		8 (24)
BMI	Healthy weight	10 (30)
	Overweight	14 (42)
	Obesity	9 (27)
Baseline LVEF %	Median (Q1, Q3)	65 (60 to 68)

Key: *ART* antiretroviral therapy, *LVEF* left ventricular ejection fraction

*Missing data on duration of ART treatment (*n* = 1), CD4 count (*n* = 18), viral load (*n* = 20), missing data on hypertension (*n* = 4)

### Post-treatment LVEF and echo parameters

**Fig. 1:**
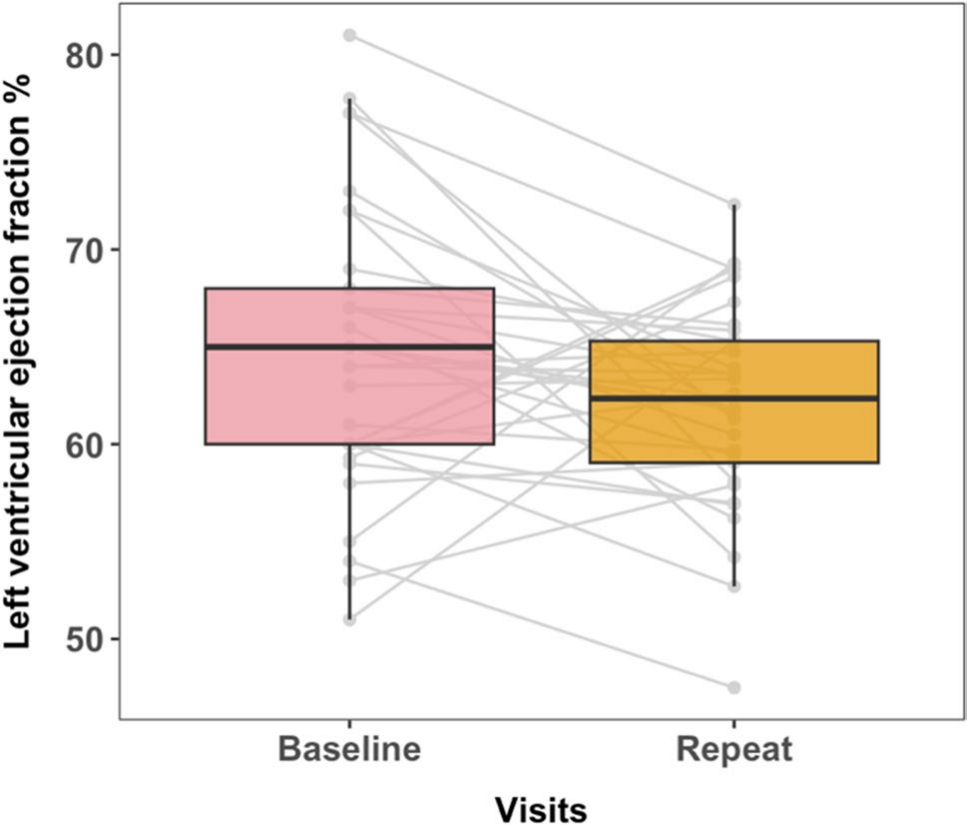
Boxplot showing changes in left ventricular ejection fraction at baseline and following treatment with doxorubicin and/or trastuzumab at least 1 year following cancer treatment initiation

Change in LVEF was assessed after a median follow-up of 2.1 years (IQR 1.8–3.1) after cancer treatment initiation. Figure 1 shows the graphical change in LVEF > 1 year after cancer therapy initiation. There was a modest but statistically significant decrease from baseline LVEF to post-treatment assessment in LVEF (−2.9%, 95%CI: −5.3 to −0.2% p = 0.038). No assessment of additional echocardiogram-derived measures were captured at baseline. Post-treatment, 12.1% had septal wall thickness ≥ 1 cm, 36.4% had relative wall thickness > 0.42, and median LV mass index was 62.4 g/m^2,^(Q1-Q3 66.1–77.0) (Supplementary Table 1). Furthermore, 36.4% of patients had lateral e’ velocity < 10 cm/s, 15.2% had septal e’ velocity < 7 cm/s, 6.5% had LA volume index > 34 ml/m^2^ but no patients had elevated LV filling pressures (E/e’ >14). Overall, 6.6% of patients had LVEF < 53%, and 3% had a left ventricular end-diastolic dimension (LVEDD) > 5.3 cm, indicative of dilation. All participants exhibited normal right ventricular systolic function, as defined by tricuspid annular plane systolic excursion (TAPSE) ≥ 1.7 cm.

### Post-treatment assessment of CV risk factors

At the time of post-treatment assessment, 33.3% had normal blood pressure, 6.1% had elevated blood pressure, 33.3% had stage 1 hypertension, and 27.3% had stage 2 hypertension. However, following cancer therapy, 30.3% reported being aware of a clinical diagnosis of hypertension. This was compared to 24.2% at baseline. 24.2% reported taking anti-hypertensive medications at the time of follow-up. Moreover, 6% reported a history of diabetes compared to none at baseline.

## Discussion

This study is the first, to our knowledge, assessing echocardiogram-derived measures of structure and function in PWH with breast cancer after doxorubicin and/or trastuzumab treatment, at a median follow-up of approximately 2 years. Our study showed that there was a modest but statistically significant LVEF decline in this population (−2.9%) following initiation of cardiotoxic cancer therapy. Our analyses also demonstrated a high prevalence of hypertension at baseline and follow-up. Importantly blood pressure measurements showed that at the time of follow-up, although 30.3% reported a clinical diagnosis of hypertension, 60.6% of patients met ACC/AHA criteria for either stage I or II hypertension based on direct blood pressure measurement. These findings suggest a high burden of cardiovascular risk and underdiagnosis of hypertension among Botswana women.

The decline in LVEF following cardiotoxic therapy is consistent with published literature in patients without HIV who received treatment with anthracycline and/or trastuzumab for breast cancer [7]. Detailed cardiac phenotyping in a US-based prospective cohort of almost 300 breast cancer patients who received doxorubicin and/or trastuzumab reported a similar decrease in LVEF of −3.6% at 1 year and − 3.8% at 3 years [7]. Although, we a priori hypothesized that PWH may experience greater declines in LVEF due to the burden of CV risk factors such as hypertension, hyperlipidemia, and diabetes mellitus [15], the LVEF decline in our cohort at 2 years following the initiation of cancer therapy was 2.9%. This finding may be reflective of a survivor bias, since eligibility in our study required patients being alive at least one year following cancer therapy initiation, in order to be enrolled for follow-up echocardiography assessment. It is also possible that there are more steep declines in LVEF during active treatment with partial recovery at 2 years, similar to longitudinal trends in non-HIV cancer cohorts [7]. Additionally, differences in LVEF assessment methods—baseline LVEF determined by cardiologist’s qualitative estimation versus follow-up LVEF measured using sonographer biplane assessment—may have limited precise comparisons.

Importantly, our study also revealed a higher prevalence of hypertension in PWH and breast cancer (30.3%) compared to baseline (24.2%). The baseline rate aligns with observations from cohort analysis of PWH in Botswana, which showed a 25% prevalence rate of hypertension [16]. In the general population, cancer therapy is known to be associated with increased risk for hypertension. This reported increase in hypertension over time warrants further study to validate these findings and guide prevention and management strategies [17]. Furthermore it is plausible that a longer follow-up could have revealed more pronounced observations, as hypertension risk is known to increase with age [14]. Most notably, applying current AHA criteria to blood pressure measurements, 60.6% met current criteria for hypertension at post-treatment assessment, higher than the previously reported rate of 36% in the general population in Botswana [18]. This is especially concerning in this population, given the established association between hypertension and elevated cardiotoxicity [19], as well as a 1.5-fold increased risk of mortality in breast cancer patients with hypertension compared to those without hypertension [20]. The discrepancy between the reported rates of clinically diagnosed hypertension (30.3%) compared with those meeting AHA criteria for hypertension (60.6%) may be reflective of a higher cut-off for hypertension diagnosis in Botswana. Finally, our results also demonstrate LV hypertrophy and increased LV mass which correlate with the rates of hypertension. These structural changes have also been observed in PWH and have been associated with higher cardiovascular mortality in some studies [21].

There are a number of limitations that may affect interpretation of our findings. First, there is a risk of survivor bias, since our sample size was limited to patients who were alive at the time of this study. Particularly given data on increased mortality in PWH and breast cancer [3, 11, 12], it is possible that patients with early deaths had worse baseline cardiovascular risk, LVEF decline, and worse cardiac function, thereby biasing our results towards the null. Additionally, our study had a small sample size. Finally, the lack of baseline assessments of echocardiogram-derived measures (aside from LVEF), including LV global longitudinal strain hindered more complete assessment of the cardiac structure and function over time. Prior research has shown that doxorubicin results in early and persistent abnormalities in 3D strain (global circumferential, longitudinal, principal strain) [22], however these were not captured at baseline or in follow-up in this pilot study. Future research should consider establishing a prospective cohort with detailed cardiovascular phenotyping, functional imaging and circulating biomarker assessments to provide more insight on longitudinal CV risk in this population. Notably, our study has several strengths. We demonstrated a high cardiovascular risk burden among PWH and breast cancer who received cardiotoxic cancer therapy. Additionally, we were able to complete detailed cardiac phenotyping in PWH following cancer treatment.

## Conclusion

Widespread ART utilization has led to longevity in people with HIV. Among those with HIV and breast cancer, this study shows a significant decline in left ventricular systolic function, and an increased burden in cardiovascular risk factors such as hypertension after cancer therapy. Further studies are needed to better understand the cardiovascular risk profile, and characterize changes in cardiac function and structure following cardiotoxic cancer therapy. Our study suggests an elevated cardiovascular risk in PWH and breast cancer, and highlights an important need for enhanced cardiovascular surveillance for this growing population.

## Supplementary information

Additional file 1. Supplementary Figure 1: Flow diagram of patient selection into the cohort.

Additional file 2. Supplementary Table 1: Summary of structure, function, and VA coupling following treatment with doxorubicin and/or trastuzumab. Supplementary Table 2: Cardiovascular risks and use of cardiac medications following treatment with doxorubicin and/or trastuzumab.

## Data Availability

Data is available upon reasonable request and subject to IRB regulations by the Human Resource Development Council, Botswana.
