# Supplementary material for: Cardiovascular risk factors and cardiac dysfunction in people with HIV and breast cancer: an observational cohort study in Botswana
Source: Cardiooncology. 2026 Jan 22;12:9. doi: 10.1186/s40959-025-00417-3 (PMC12829206; doi:10.1186/s40959-025-00417-3)

Supplementary Figure:

Supplementary Figure 1: Flow diagram of patient selection into the cohort


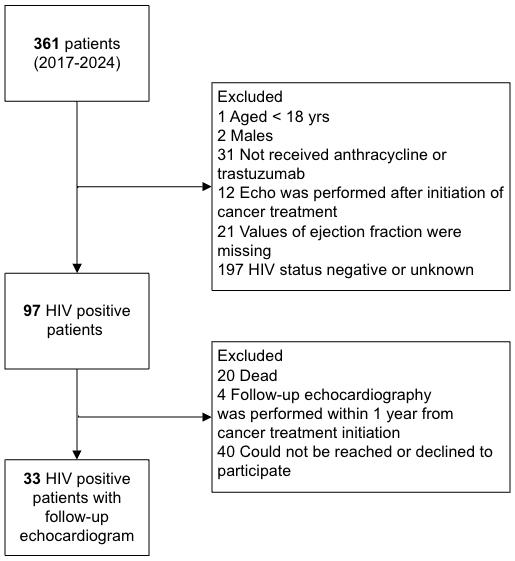

Supplement: Supplementary file 1 — Additional file 1. Supplementary Figure 1: Flow diagram of patient selection into the cohort. [file 40959_2025_417_MOESM1_ESM.docx]
