# Supplementary material for: Cardiovascular risk factors and cardiac dysfunction in people with HIV and breast cancer: an observational cohort study in Botswana
Source: Cardiooncology. 2026 Jan 22;12:9. doi: 10.1186/s40959-025-00417-3 (PMC12829206; doi:10.1186/s40959-025-00417-3)
Supplement: Supplementary file 2 — Additional file 2. Supplementary Table 1: Summary of structure, function, and VA coupling following treatment with doxorubicin and/or trastuzumab. Supplementary Table 2: Cardiovascular risks and use of cardiac medications following treatment with doxorubicin and/or trastuzumab. [file 40959_2025_417_MOESM2_ESM.docx]

Supplementary Tables:

Supplementary Table 1: Summary of structure, function, and VA coupling following treatment with doxorubicin and/or trastuzumab

|  |  | n (%) |
| --- | --- | --- |
| Right atrial pressure, mmHg | 3-5 mmHg | 33 (100.0) |
|  | 5-8mmHg | 0 (0.0) |
|  | 8-15mmHg | 0 (0.0) |
| Pulmonary regurgitation | None | 17 (51.5) |
|  | Trivial | 11 (33.3) |
|  | Mild | 5 (15.2) |
|  | Mild to Moderate | 0 (0.0) |
| Tricuspid regurgitation | None | 13 (40.6) |
|  | Trivial | 5 (15.6) |
|  | Mild | 14 (43.8) |
|  | Mild to Moderate | 0 (0.0) |
|  | Moderate | 0 (0.0) |
| Mitral regurgitation | None | 14 (42.4) |
|  | Trivial | 9 (27.3) |
|  | Mild | 9 (27.3) |
|  | Mild to Moderate | 1 (3.0) |
| LV mass index, g/m^2^ | Median (Q1, Q3) | 62.4 (57.0, 66.1) |
| LV septal wall thickness, cm | ≥ 1 | 4 (12.1) |
| LV posterior wall thickness, cm | ≥ 1 | 1 (3.0) |
| Relative wall thickness | > 0.42 | 12 (36.4) |
| Lateral e’ velocity, cm/s | < 10 cm/s | 12 (36.4) |
| Septal e’ velocity, cm/s | < 7 cm/s | 5 (15.2) |
| LA volume index, ml/m^2^ | > 34 ml/m^2 | 2 (6.5) |
| LV internal diastolic diameter | > 5.3 cm | 1 (3.0) |
| LVEF category | <50 | 1 (3.0) |
| LVEF, % | <53 | 2 (6.6) |
| LVEF, % | < 55 | 3 (9.0) |
| E/e’ | <=14 | 33 (100.0) |
| TAPSE | >1.7cm | 33 (100.0) |

Data are N (%), median (Q1, Q3)

Supplementary Table 2: Cardiovascular risks and use of cardiac medications following treatment with doxorubicin and/or trastuzumab

|  | N (%) |
| --- | --- |
| Measured blood pressure categories |  |
| Normal Blood Pressure | 11 (33.3) |
| Elevated Blood Pressure | 2 (6.1) |
| Hypertension Stage 1 | 11 (33.3) |
| Hypertension Stage 2 | 9 (27.3) |
| ACE inhibitor or angiotensin receptor blocker | 2 (6.1) |
| Beta blocker | 1 (3.0) |
| Calcium channel blocker | 4 (12.1) |
| Diuretics | 6 (18.2) |
| Any antihypertensive | 8 (24.2) |
| Statin | 1 (3.0) |
| Hypertension at follow-up | 10 (30.3) |
| Diabetes at follow-up | 2 (6.0) |
